# Antioxidant Defenses in the Human Eye: A Focus on Metallothioneins

**DOI:** 10.3390/antiox10010089

**Published:** 2021-01-11

**Authors:** Ana Álvarez-Barrios, Lydia Álvarez, Montserrat García, Enol Artime, Rosario Pereiro, Héctor González-Iglesias

**Affiliations:** 1 Instituto Universitario Fernández-Vega (Fundación de Investigación Oftalmológica, Universidad de Oviedo), 33012 Oviedo, Spain; ana.alvarezbarrios1@gmail.com (A.Á.-B.); l.alvarez@fio.as (L.Á.); mgarciadiaz@fio.as (M.G.); enol.artime@fio.as (E.A.); mrpereiro@uniovi.es (R.P.); 2 Department of Physical and Analytical Chemistry, Faculty of Chemistry, University of Oviedo, Julián Clavería, 8, 33006 Oviedo, Spain; 3 Instituto Oftalmológico Fernández-Vega, Avda. Dres. Fernández-Vega, 34, 33012 Oviedo, Spain

**Keywords:** eye, oxidative stress, natural barriers, ocular diseases, antioxidants, metallothioneins

## Abstract

The human eye, the highly specialized organ of vision, is greatly influenced by oxidants of endogenous and exogenous origin. Oxidative stress affects all structures of the human eye with special emphasis on the ocular surface, the lens, the retina and its retinal pigment epithelium, which are considered natural barriers of antioxidant protection, contributing to the onset and/or progression of eye diseases. These ocular structures contain a complex antioxidant defense system slightly different along the eye depending on cell tissue. In addition to widely studied enzymatic antioxidants, including superoxide dismutase, glutathione peroxidase, catalase, peroxiredoxins and selenoproteins, inter alia, metallothioneins (MTs) are considered antioxidant proteins of growing interest with further cell-mediated functions. This family of cysteine rich and low molecular mass proteins captures and neutralizes free radicals in a redox-dependent mechanism involving zinc binding and release. The state of the art of MTs, including the isoforms classification, the main functions described to date, the Zn-MT redox cycle as antioxidant defense system, and the antioxidant activity of Zn-MTs in the ocular surface, lens, retina and its retinal pigment epithelium, dependent on the number of occupied zinc-binding sites, will be comprehensively reviewed.

## 1. Introduction

The eye is subjected to a highly oxidative environment due to its intense exposure to light, its robust metabolic activity, and its high oxygen tension. Considering that solar ultraviolet (UV) radiation is the major environmental inducer of oxidant reactive species formation, the eye has developed a complex protection system against oxidative damage organized in three levels: (1) Prevention of free radical yield by pigments capable of absorbing and filtering light; (2) detoxification of free radicals by enzymatic or non-enzymatic antioxidants; and (3) repairment systems of oxidized biomolecules [1]. In the following sections, the antioxidant enzymes that are part of the defense system of the human eye will be discussed, with a particular focus on the redox system zinc-metallothioneins.

### 1.1. The Human Eye

**Figure 1:**
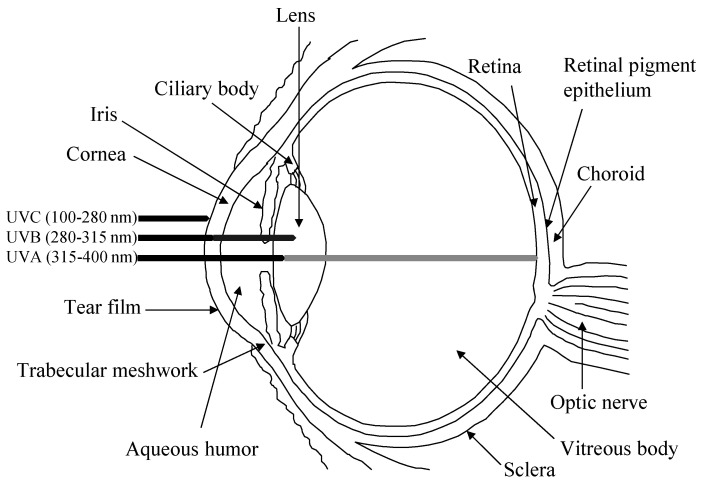
Schematic view of the human eye anatomy and representation of the absorption of UV radiation by the main ocular barriers.

The human eye is a highly specialized organ of the visual system consisting of three primary layers surrounding the bulk of the ocular globe (Figure 1). The external part is the fibrous tunic, containing the sclera and cornea, the middle part is the uvea or vascular tunic composed of iris, ciliary body (CB), and choroid, and the inner part is the neural layer formed by the retina and its retinal pigment epithelium (RPE). These coats surround the lens and the transparent media, namely anterior chamber with aqueous humor and vitreous body [1]. Oxidative stress affects all the structures of the eye, specifically its antioxidant natural barriers formed by the ocular surface (i.e., cornea and the anterior part of the sclera), the lens and retina.

The cornea and sclera form a protective envelope that protects the ocular tissues and also provides structural support. The transparency of the cornea and its refractive power are among the most important properties, presenting a tough physical barrier to trauma and infection. The cornea has five different layers: The corneal epithelium, a stratified, squamous, non-keratinized epithelium; the anterior limiting membrane or Bowman’s layer, a modified acellular region of the stroma; the corneal stroma, a connective tissue composed of collagenous lamellae, keratocytes and modified fibroblasts; posterior limiting lamina or Descemet’s membrane; and corneal endothelium, a single layer of hexagonal cells. The lens, also responsible for light refraction and focus over the retina, possesses less refractive power than the cornea. This transparent body is composed of long-fibre like cells and epithelial cells highly organized and tightly packed, enclosed in an elastic collagenous capsule that confers its ability to change shape by the accommodation process. The retina is a light-sensitive tissue that lines the inner surface of the posterior segment of the eye, whose main function is to detect light, convert photochemical energy into neural signals, and transmit the signals to the visual cortex of the brain. The retina consists of an inner multilayer of neurosensory cells and an outer single neuroepithelium, the RPE. The neuroretina comprises the nerve fiber layer, the ganglion cell layer, the inner plexiform layer, the inner nuclear layer (INL), the outer plexiform layer and the outer nuclear layer (ONL), formed by different cell types including vascular endothelial, pericytes, glial, microglia, ganglion, horizontal, amacrine, bipolar, and photoreceptors. In addition, the RPE is a pigmented cell monolayer that nourishes and maintains the adhesion of the neurosensory retina and reduces light scattering within the eye [2].

### 1.2. Reactive Oxygen Species within the Eye

Oxidative stress occurs from formation of multiple reactive oxygen species (ROS), including superoxide, hydrogen peroxide, and hydroxyl radicals, which promote free radical production. These oxygen-containing free radicals are produced as natural subproducts of the normal metabolism of oxygen, and have important roles in cell signaling and homeostasis. An imbalance of ROS production and the capability of the eye to counteract these free radicals causes oxidative stress that may be amplified by a continuing cycle of metabolic stress leading to increased free radical production. It compromises the antioxidant capacity of free radical scavenger systems and triggers cell damage and death. This intracellular disequilibrium results in the onset and/or progression of ocular diseases, which is exacerbated during long-term oxidative stress exposure and ageing [3].

While exogenous ROS generators include UV light exposure, viral infection, chemical insults and drug intake, among others, endogenous ROS are mainly produced in the mitochondria due to cellular respiration [4]. UV light, one of the main factors of ROS production triggering oxidative damage, consists of radiation in different wavelengths: UVA (315–400 nm), UVB (280–315 nm) and UVC (100–280 nm). Whilst the cornea and lens absorb UVC radiation and most UVB, a small part of UVA radiation reaches the retina-RPE system (see Figure 1). This continuous oxidative damage plays a key role in the onset of age-related eye diseases affecting the cornea, lens and retina-RPE [5]. In the cornea, UV light producing ROS interferes with epithelial cells proliferation and reduces epithelial thickness, which may promote Fuchs’ endothelial dystrophy. This corneal disorder associated to oxidative stress leads to degeneration of endothelium causing stromal and epithelial edema resulting in visual acuity reduction. This corneal dystrophy is characterized by the loss of hexagonal shape and cell density of endothelial cells and formation of excrescences of Descemet’s membrane called guttae [6]. Besides, the lack of blood vessels in the lens exacerbates oxidative stress, increasing the risk of its opacification and resulting in cataract formation, which is one the most common causes of blindness worldwide [7,8,9]. Furthermore, the high oxygen consumption rate of retinal cells due to cellular respiration contributes synergistically with UV light and vascular flux defects to the pathogenesis of retinal diseases, including glaucoma, age-related macular degeneration (AMD), diabetic retinopathy and retinitis pigmentosa, among others [10]. During glaucoma, a neuropathy characterized by the abnormal increase of intraocular pressure that brings to degeneration of retinal ganglion cells (RGCs) causing irreversible blindness, a prolonged imbalance of oxidative species and antioxidants is considered a significant risk factor where high intraocular pressure stimulates in a feedback process the production of ROS, inducing RGCs to autophagy and apoptosis [11]. Moreover, the continuous exposure of the RPE to light energy, the oxygen-rich environment and the high metabolic activity of this neuroepithelial monolayer provide an ideal framework for the formation of ROS with the potential to damage proteins, DNA and lipids, resulting in AMD, a neurodegenerative disease, characterized by the irreversible damage in the macula and the formation of extracellular deposits between RPE and Bruch’s membrane (a modified connective tissue layer), called drusen [12]. Finally, the microvascular disease diabetic retinopathy is a complication of diabetes mellitus, caused by high mitochondrial ROS production, due to hyperglycemia. This results in capillaries cell damage and the release of plasma and erythrocytes in the retina causing edema and stimulating retinal angiogenesis [13].

## 2. Antioxidant Defense Systems in the Eye

**Figure 2:**
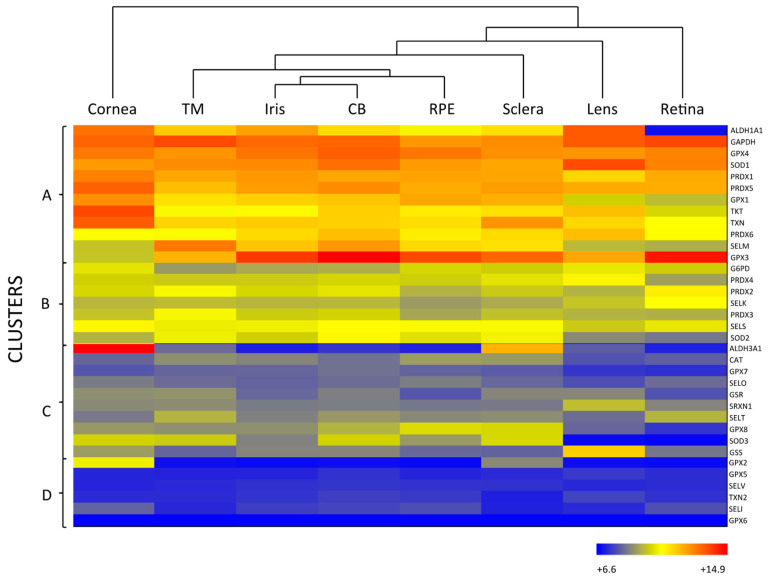
Hierarchical cluster and heat map (whole-genome expression microarrays) of the main antioxidant enzymes in human ocular tissues. The row at the top shows the clustering information in the form of a dendogram and the similarity relationships among the genes and tissues: cornea (n = 11), trabecular meshwork (TM; n = 9), CB (n = 12), sclera (n = 7), iris (11), RPE (n = 8), retina (n = 12) and lens (n = 10). The column at the left of the heat map shows four clusters (A to D), each with antioxidant enzymes expressed at different abundance. Mean values of 6.6–14.9 from biological replicas per tissue are indicated according to the log2 scale, in arbitrary units, depicted at the bottom. Hierarchical cluster and heat maps were created using Illumina BeadChip array platform (HumanHT-12 v4.0 Expression BeadChip Kit, Illumina, San Diego, CA, USA) and ArrayStar software, version 4 (DNASTAR, Inc, Madison, WI, USA) according to Alvarez et al., 2012 [15]. n: number of samples per tissue.

As mentioned above, the eye is constantly exposed to both exogenous and endogenous sources of oxidants, which put the cells under continuous pressure from oxidative stress. The eye relies on a complex antioxidant defense system to keep free radicals under control and maintain correct physiological functions [5]. Defense against oxidative stress is slightly different across the eye, matching with its structural complexity and accounting for the different sources of ROS threatening each region. For the purpose of this review, we have divided the eye into three natural barriers of defense: (1) The ocular surface, composed by the tear film, cornea, anterior sclera and aqueous humor; (2) the lens; and (3) the retina (neurosensory retina and RPE). Although it is out of the scope of this review, it should be noted the potential antioxidant role of the vitreous body, a body fluid of gel nature filling the posterior pole between the lens and the retina, filtering infrared light, has been recently reviewed [14]. Figure 2 shows the hierarchical ranking and clustering of enzymatic antioxidant genes present in the human eye, based on whole-genome expression microarray analysis by Alvarez et al., 2012 [15]. Four clusters could be distinguished (A, B, C and D), each containing genes expressed at levels ranging from highly abundant (i.e., cluster A) to moderate (i.e., cluster B and C), and almost absent (cluster D), which will be further discussed with respect to their ocular tissue antioxidant barrier function.

The antioxidant defense system of any cell is composed of a vast variety of compounds, including both enzymatic and non-enzymatic molecules. Non-enzymatic antioxidants are water-soluble (e.g., ascorbic acid and glutathione) or fat-soluble (e.g., carotenoids and vitamins A and E) molecules with relevant roles, not only in antioxidant protection, but also in UV-light absorption [16,17], by which other scientific papers are referenced [18,19,20]. In relation to enzymatic antioxidants, primary and secondary enzymatic defenses can be distinguished. Primary enzymatic antioxidants consist of enzymes that act at the root of the oxidative propagation chain, preventing the formation of new free radicals by removing precursors or inhibiting catalysts. These primary antioxidants include enzymes like superoxide dismutase (SOD), glutathione peroxidase (GPX), catalase (CAT) and peroxiredoxins (PRDXs). By comparison, secondary enzymatic antioxidants have preventive and supportive roles in antioxidant protection and may tackle different processes like metal deactivation, regeneration of primary antioxidants and maintenance of the cellular reducing power. Secondary antioxidants include metabolic enzymes like transketolase (TKT), glucose-6-phosphate dehydrogenase (G6PD) and glyceraldehyde-3-phosphate dehydrogenase (GAPDH); glutathione-redox cycle enzymes like glutathione reductase (GSR) and glutathione synthetase (GSS); selenoproteins (SELM, SELO, SELT, SELK, SELS, SELV and SELI) and thioredoxin-related enzymes like thioredoxin (TXN) and sulfiredoxin (SRXN) [21,22]. Both primary and secondary antioxidants are important constituents of the ocular surface, the lens, and the retina of the human eye.

### 2.1. Enzymatic Antioxidants of the Ocular Surface

As the outermost region of the eye, the ocular surface comprised of the cornea, sclera, tear film and aqueous humor, is directly exposed to environmental damaging agents. Considering that UV-light radiation is the major source of ROS among these environmental stressors, the ocular surface has a crucial function in UV-light filtering and absorption, protecting the inner ocular tissues from oxidative damage and constituting the first barrier of defense against oxidants [19,20]. However, UV-light absorption makes the cells of the cornea and anterior sclera themselves vulnerable to the effects of ROS, thus, requiring a robust antioxidant defense system optimized for both their function and protection [23].

#### 2.1.1. Primary Antioxidant Enzymes

SOD catalyzes the dismutation of superoxide anion (O_2_·) to the less toxic free radical hydrogen peroxide (H_2_O_2_), being the only antioxidant, capable of reacting with O_2_·, and thus, often regarded as the most important primary antioxidant of the corneal antioxidant defense system [5]. Humans have three isoforms of SOD, namely SOD1, SOD2 and SOD3, that differ in their location (SOD1 is located in the cytosol, SOD2 in the mitochondria and SOD3 in the extracellular space) and cofactors (SOD1 and SOD3 use Cu and Zn, while SOD2 uses Mn) [16]. SOD isoforms also have different sensitivity to UV-light and oxidative stress, given that SOD1 is easily down-regulated by UVB, while SOD2 can be moderately induced under light-induced oxidative stress [24]. Due to their differences, some authors suggest that SOD isoforms perform different functions in the eye physiology. This hypothesis is supported by the fact that knockout animals show significantly different phenotypes depending on the isoform inactivated [19,25]. According to Figure 2, all three SOD isoforms are present in the ocular surface, being SOD1 the most expressed isoform in all the considered eye tissues, including the cornea. While SOD2 is a minor isoform of SOD in the human cornea, it is abundant in other mammalian ocular tissues and has gained interest because of its antioxidant function within the mitochondria, and its role in the prevention of apoptosis initiated by mitochondrial damage [26,27].

Although H_2_O_2_ is more stable than O_2_·, it can diffuse across hydrophobic membranes and generate other ROS such as OH^-^ by reacting with metal ions. Therefore, H_2_O_2_ poses an important threat to cell function and numerous enzymes are committed to its detoxification, i.e., CAT, GPX and PRDX [28,29]. CAT is a heme-containing enzyme that catalyzes the dismutation of H_2_O_2_ into O_2_ and H_2_O using Mn as a cofactor. It also protects SOD from inactivation and may be directly involved in UVB-light filtering. CAT is ubiquitously expressed in mammalian cells, predominantly in peroxisomes [19,25], and the microarray analysis data depicted in Figure 2 suggest that CAT is expressed at low levels both in the cornea and sclera.

GPX catalyzes the same reaction as CAT, reducing H_2_O_2_ to H_2_O, but using reduced glutathione (GSH) as an electron donor. GPX activity depends on the regeneration of GSH, which is carried out by enzymes like GSR and GSS that produce GSH by reducing oxidized glutathione (glutathione disulfide, GSSG) or by de novo synthesizing it, respectively [5,19]. Eight isoforms of GPX are known in humans and five of them (GPX1–4 and GPX6) require Se as cofactor [25]. Like other selenoproteins, GPX family follows a hierarchical order for Se supply, being GPX1 and GPX4 the most preferentially isoforms [30]. Six isoforms of GPX are expressed in the ocular surface, with preferential presence of GPX1 and GPX4, in addition to GPX3 in the sclera (Figure 2). In comparing GPX and CAT expression within the eye, it seems that GPX rather than CAT may predominantly perform H_2_O_2_ decomposition in the cornea and sclera. Conversely, CAT may gain importance over the GSH redox system when H_2_O_2_ concentration increases [20,31].

PRDX is the third type of enzyme able to reduce and detoxify H_2_O_2_ using its redox active structural cysteines without the need of specific cofactors [14]. Six PRDX isoforms are expressed in humans, namely PRDX1-6, all localized both in the cornea and the sclera. PRDX may also have chaperone-like and redox-signaling properties, which link them to inflammation and aging processes [28,32].

#### 2.1.2. Secondary Antioxidant Enzymes

A big part of the secondary antioxidant enzymes of the cornea belongs to a group of water-soluble proteins formerly called crystallins, which include very diverse proteins, like chaperones, metabolic enzymes, and members of the heat-shock family. Berzelius originally coined the name in 1830 to describe highly abundant proteins present in the bovine crystal-clear lens [33]. Later, it was found that crystallins are also present in the cornea and, less abundantly, in some outer-eye tissues [34]. In the eye, their main function is to provide the lens and cornea with the required transparency and refractive index to filter and focus the light adequately. These proteins are carefully packed to minimize variations in concentration and to create an appropriate concentration gradient across the eye [33]. Developmental modulators and environmental factors tightly regulate crystallins genes in order to create such concentration gradient [35]. During aging and/or post-translational modifications, crystallins are prone to aggregate, which disturbs their concentration pattern and the light focusing capacity of the eye, even possibly leading to cataracts exacerbated by oxidative stress [33,36,37]. Interestingly, crystallins in the eye also have additional functions involved in UV-light filtering, protein folding and ROS detoxification [24]. The multifunctionality of crystallins is caused by a phenomenon called “gene sharing”, which occurs when a gene codifies a protein that has remarkably different functions depending on its concentration and expression pattern [33]. This finding expanded the knowledge about crystallins by including enzymes that were long thought to only be involved in cellular processes, such as metabolism and stress response [35].

One of such called enzymatic crystallins is the aldehyde dehydrogenase (ALDH) family, which regulates the metabolism of aldehydes and has a function as crystallins, albeit only ALDH1A1 and ALDH3A1 are expressed in some tissues of the eye [38]. In the cornea, ALDH family is the most abundant secondary antioxidant, especially the isoform 3A1 (ALDH3A1), which is the most expressed corneal antioxidant enzyme (Figure 2) accounting for 5 to 50% of the total water-soluble protein fraction of the corneal epithelium in mammalian species [39]. ALDH superfamily catalyzes the NAD(P)^+^-dependent oxidation of a wide range of endogenous and exogenous toxic aldehydes and participates in the antioxidant defense by directly scavenging ROS and indirectly producing NADPH. Although, ALDH3A1 is considered a cytosolic protein, it has been also found in the cellular nucleus, suggesting that it may also protect DNA from oxidative damage [40]. While ALDH3A1 is almost exclusively expressed in the cornea and sclera, ALDH1A1 is present in all the eye tissues with the exception of the retina, being highly expressed in the cornea ahead of other abundant enzymes like SOD (Figure 2). These findings support the idea that the cornea requires a higher number of antioxidants than other parts of the eye to maintain its UV-light filtering function.

The enzymes TKT and GAPDH are found in great abundance in the cornea—hence, they are recognized as being crystallins—highlighting that TKT is the second most abundant secondary antioxidant enzyme just behind ALDH3A1. TKT metabolizes glycolytic products as part of the pentose phosphate pathway (PPP) and GAPDH is an enzyme involved in the glycolysis [41]. PPP and glycolysis are closely associated routes that generate ribose-5-phosphate, the essential building block for RNA synthesis, and ATP via glucose oxidation, respectively. Both pathways also generate NADPH, which is the main reducing agent of the cell, so enzymes like TKT and GAPDH serve an additional antioxidant role by promoting NADPH production [24].

It should be noted that the cornea counts with other crystallins that are non-enzymatic, such as chaperones, whose activity is fundamental to prevent protein aggregation and to maintain the transparency of the cornea [34]. In some instances, chaperones also prevent oxidative stress by conserving the structure of antioxidant enzymes, e.g., αB-crystallin reduces aggregation of mutant SOD-1 in familial amyotrophic lateral sclerosis and secures its antioxidant activity [42].

### 2.2. Enzymatic Antioxidants of the Lens

Together with the cornea, the lens is responsible for focusing the incident light into the retina, as well as filtering harmful UV radiation to protect the inner tissues, constituting a second barrier of antioxidant defense. The UV radiation that reaches the lens is greatly composed of UVA light, since the cornea absorbs UVC and most UVB but only a small fraction of UVA (see Figure 1). UVA radiation has been shown to be more effective at causing oxidative damage than any other UV radiation, putting the lens at a higher risk of oxidative stress induction since its absorption may cause damage to the cells [43,44]. Also, lens metabolism occurs mainly in the epithelium, while the synthesis of new proteins ceases with lens fiber cell formation, making the lens especially susceptible to the accumulation of oxidative damage, and accounts for its different antioxidant needs in comparison with the aforementioned cornea [28,45,46].

The lens contains high levels of GPX and SOD [1]. Superoxide dismutases are one of the main antioxidant enzymes of the ocular lens, where they show a clear distribution consisting of high levels of protein in the anterior layers of the lens (i.e., lens capsule and epithelial cells) and low levels in the posterior layers (i.e., cortex and nucleus composed of lens fibers) [47]. Although SOD2 and SOD3 may not be present at RNA level (Figure 2). The isoform SOD1 contributes about a 90% to the total SOD activity in the lens [48], whilst the overall SOD activity significantly decreases with age and is probably caused by the life-long accumulation of inhibitory modifications, as protein turnover is almost non-existent in the cortex and the nucleus. In contrast, the activity of SOD in the cornea does not decline to such extent during aging, reflecting the differences in protein synthesis and turnover between these eye tissues [47,49].

Low CAT RNA-expression levels are observed in the whole lens (Figure 2). Some studies show that CAT is more concentrated in the periphery of the lens epithelium, where cells may require a stronger defense system [50]. Recent studies have been centered on the signaling function of H_2_O_2_ in the cell, which has been shown to occur by transient bursts of oxidants that inactivate both CAT and PRDXs [51,52]. However, it is most likely that daily H_2_O_2_ decomposition in the lens is carried out by PRDXs and GPX, according to their raised abundance in the tissue, and that CAT is more active against chronic exposure to H_2_O_2_ similarly to its function in the cornea [18,28]. All PRDX isoforms are, to a greater or lesser extent, expressed in the lens, as well as in the rest of the ocular tissues, suggesting a distinct and specific role in eye physiology for each of them (Figure 2) [53].

The glutathione redox system is especially important in the lens, since its high protein content calls for a strict redox control on thiol groups to avoid protein aggregation and this system can fulfill such function [54]. According to Figure 2, among the eight isoforms of human GPX, the lens mostly expresses GPX1, GPX3 and GPX4. GPX1 is the main intracellular isoform of GPX and its deficiency has been linked with membrane damage in the lenticular nucleus. Neither the epithelium nor the cortex of the lens is significantly affected by GPX1 loss, probably due to the abundance of other compensating antioxidants [55]. In another study, GPX3 is secreted and appears in the extracellular space, while GPX4, a mediator in the non-apoptotic and iron-dependent programmed cell death pathway that produces a rapid release of ROS (i.e., ferroptosis) is coupled with cell membranes [56].

As discussed in the previous section, crystallins are extremely important to the lens due to their dual role in antioxidant protection and light refraction. Among the enzymatic crystallins, members of the ALDH superfamily are the most abundant in the lens, specifically the isoform ALDH1A1, just behind SOD1 levels [38]. In contrast to the cornea, ALDH activity in the lens is carried out entirely by ALDH1A1 and not ALDH3A1. Although, in some species, ALDH1A1 has been described in the cornea at even greater levels than ALDH3A1, the later has never been reported to have significant abundance in the lens [44,57]. This suggests that ALDH1A1 has an important function both in the cornea and the lens, which could be related to the detoxification of reactive aldehydes. Meanwhile, ALDH3A1 may have a bigger role in UV radiation filtering than aldehyde detoxification, since by filtering light in the cornea it also indirectly protects the underlying lens [16]. ALDH1A1 has a heterogeneous distribution throughout the lens, being more concentrated in the epithelial cells and the cortex than in the nucleus, presumably because of its bigger exposure to free radicals coming from the aqueous humor [57].

Finally, metabolic enzymes, involved in NADPH regeneration, like GAPDH and TKT, are also an important part of the lens antioxidant defense system. Differential expression of TKT in the lens and cornea (Figure 2) may be the result of different gene regulatory mechanisms. It is suggested that crystallin gene expression in the lens is mostly regulated by tissue- and developmental-specific transcription factors, while environmental agents may play a more determining role in the expression profile of the cornea [58].

### 2.3. Enzymatic Antioxidants of the Retina

The retina is the innermost part of the eye and has a crucial role in vision, being the structure that transforms light energy into electrical outputs that are transmitted to the brain. Due to its function in vision, the retina has the highest oxygen consumption rate per kg of the body, and thus, is particularly exposed to ROS of endogenous origin [20]. In addition, photoreceptors contain high concentrations of polyunsaturated lipids that are easily oxidized and further increase the susceptibility of the retina to oxidative stress. Apart from endogenous sources, ROS in the retina can also arise from short-wavelength light that is not filtered by the first and second barriers of defense, specifically UVA and blue visible light. The RPE decreases light scatter and protects against oxidative stress generated by photooxidation. However, light-induced oxidative stress risk increases with age due to antioxidant deficiency and the accumulation of oxidative agents [59], contributing to the onset of age-related eye diseases affecting the retina. The macular region, located near the center of the retina-RPE, is particularly susceptible to light damage, and contains yellow pigments (carotenoids) that reduce glare from short wavelength blue light. These carotenoids, referred to xanthophyll macular pigments, also have an antioxidant role particularly at low levels of oxygenation. In addition, the pigmented RPE contains high amounts of melanin, which in the reduced form is a very effective free radical scavenger [1].

Primary enzymes like SOD, GPX, CAT and PRDXs are the main components of the enzymatic antioxidant defense system of the retina-RPE. The CAT concentration in the retina increases upon light exposure, but its levels do not change during prolonged exposure times. In such cases, SOD and GPX may take over CAT for antioxidant protection. In fact, CAT activity has been found to decrease with age and during age-related pathologies like AMD, while SOD does not show a clear correlation with aging [20]. However, the SOD distribution across the retina seems to change during aging [60], and recently, it was reported that SOD2 isoform experiences an age-related increase in the neural retina and more prominently in the macular region. Although, it should be noted that SOD2 levels in the retina are low in comparison to SOD1 and other antioxidant enzymes [59].

Interestingly, GPX isoform composition in the retina differs from that of the cornea and lens, being GPX3 the most abundant isoform followed by GPX4. Moreover, the GPX3 is the enzymatic antioxidant with the highest expression in the retina (Figure 2). GPX3 is present in the extracellular space, where it may protect the cell surface and basal membranes [61]. It has been reported that GPX3 is present in high levels in the aqueous humor and ciliary body of human and bovine eyes, but its presence and role in the retina has not been deeply studied [62,63]. It should be noted that GPX1 is abundantly expressed in the retina of the immature eye, likely acting as an early defense mechanism [64]. During aging, a similar pattern of expression to SOD has been described for GPX, with up-regulated levels in the photoreceptor outer segments, down-regulated levels on the peripheral retina and increased concentration upon light exposure [65]. In a recent work, the enzyme glyoxalase 1 prevented induced oxidative stress damage through the detoxification of methylglyoxalm, whose excess inactivates GPx and SOD, which may be involved in the etiopathogenesis of retinitis pigmentosa [66].

The PRDX family also contributes to the detoxification of H_2_O_2_ in the retina, although it does not show the same relevance as SOD and GPX enzymes. PRDX may complement the SOD/GPX H_2_O_2_-detoxification system by acting as redox sensors in the retina, since under oxidative stress they can become hyperoxidized and prompt redox signaling cascades, as well as function as protein chaperones. PRDX show a distinct distribution in the neural retina depending on the cellular type, where PRDX1 is mainly associated with cells in the inner nuclear layer and cone photoreceptors and PRDX5 is ubiquitously expressed in the mitochondria and sometimes found in peroxisomes, the cytoplasm and the nucleus [67].

Other antioxidants like GAPDH and some selenoproteins work in a supportive role to SOD, GPX, PRDX and CAT enzymes. Specifically, GAPDH is the secondary antioxidant most expressed in the retina due to its role in glycolysis and NADPH generation and contributes to the maintenance of the cellular reducing power under oxidative stress. Interestingly up to twenty crystallins have been described in the retina, most of them sparsely expressed and carrying out different functions than those they have in the cornea and lens. It is plausible that some crystallins interact with the cytoskeleton of retinal cells to maintain its structural integrity [68]. The overexpression of crystallins has been found to protect the RPE from oxidative stress, since some of them act as heat-shock proteins and metabolic enzymes involved in NADPH generation [69].

## 3. Metallothionein Antioxidant System

**Figure 3:**
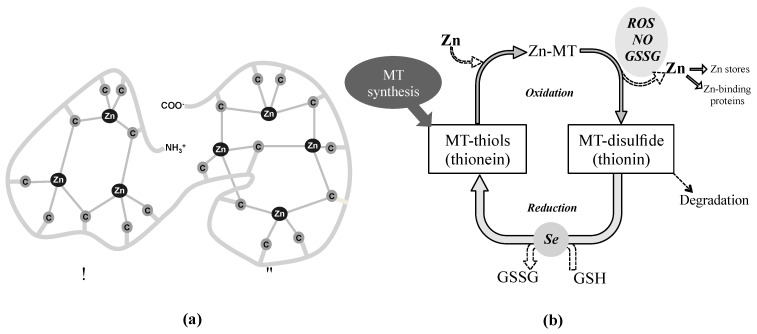
(**a**) Mammalian MTs contain 60–68 amino acids, 20 of which are cysteines that bind seven divalent metal ions in two metal/thiolate clusters, the α- and β-metal-binding domains. Eleven cysteines bind four zinc ions in the C-terminal α-cluster, whereas nine cysteines bind three zinc ions in the N-terminal β-cluster. (**b**) Zinc–metallothionein redox cycle: zinc bound to MT (Zn–MT) is released under physiological oxidative conditions, forming MT-disulfide (thionin), process boosted by free radicals derived from nitric oxide, ROS and oxidized glutathione. MT-disulfide may be degraded or reduced to MT-thiol (thionein) in the presence of a selenium-derived catalyst. MT-thiol binds zinc to form the thermodynamically stable Zn–MT system. Adapted from Gonzalez-Iglesias et al., 2014 [138].

One of the antioxidant systems of growing interest in the eye is constituted by the metallothioneins (MTs) that capture and neutralize free radicals through their cysteine sulfur ligands serving as zinc-ion donors in a redox-dependent fashion. Although it is not classically considered an enzymatic antioxidant system per se, its redox activity relies on the redox-dependent zinc release. The family of MTs is a group of cysteine rich and metal-binding low molecular mass proteins (6–7 kDa) present in all eukaryotes and many prokaryotes [70]. MT molecule is composed of two structural domains with a high content of sulfur and metals, forming metal-thiolate clusters through the covalent binding of metal atoms with the sulfhydryl cysteine residues. As shown Figure 3 (panel a), the N-terminal part of the peptide (β-domain) has three binding sites for divalent ions and the C-terminal part (α-domain) can bind up four divalent metal ions [71]. These proteins bind mainly Zn, Cu, Cd and Hg, while in mammals, under physiological conditions, MTs mostly contain zinc divalent ions, which can be displaced by copper or cadmium if they are in excess [72,73]. The coexistence of stable, partially metalated MTs along with the apo form of the protein and the fully-metalated protein indicate a non-cooperative metalation mechanism [74,75,76].

### 3.1. General Properties

#### 3.1.1. Classification

In humans, the MT family consists of four groups designated as MT1 to MT4, containing eleven isoforms and subisoforms and codified by 11 active genes located in a cluster on chromosome 16 (*MT1A*, *MT1B*, *MT1E*, *MT1F*, *MT1G*, *MT1H*, *MT1M*, *MT1X*, *MT2A*, *MT3* and *MT4*) [77], although there are five additional genes (pseudogenes), not expressed in humans [78]. All MTs share a high degree of homology at the nucleotide and amino acid levels and retain 20 invariant metal-binding cysteinyl residues [79]. While MT1 and MT2 are present in almost all tissues, MT4 is most abundant in certain stratified tissues [80] and the expression of MT3 has been exclusively related to brain. Although this latter one is also present in heart, kidneys and reproductive organs [81,82] being even a ubiquitously expressed gene [78]. As discussed below, in the human eye, MT1 and MT2 isoforms are expressed to a greater or lesser extent in all of the ocular tissues, while MT3 expression is restricted mainly to the retina and MT4 transcript is not detected [15].

#### 3.1.2. Functions

MTs, considered multipurpose proteins, are involved in a great diversity of processes, including cellular zinc homeostasis, metal detoxification, defense against oxidative damage through free radical scavenging and neuroprotection [83].

Zinc is an integral part of up to 10% of human proteins [84], being its homeostasis essential for proper cell function and metabolism. Cellular zinc homeostasis is dependent on proteins involved in the influx and efflux of zinc from cells (zinc transporters) and storage within cells (zinc-binding proteins). MTs also function as the main intracellular zinc reservoir as one MT molecule can bind up to seven zinc ions under physiological conditions. Zinc binding and release to MTs is mainly conditioned by the cellular redox state, where oxidation of cysteine residues of MTs facilitates the zinc transfer from these proteins to a wide variety of metalloproteins and transcription factors, regulating numerous cellular processes [85,86,87,88]. In addition to zinc ions, which are considered the main binding partner of apo-protein, MTs bind with high affinity other metal or heavy metal ions (e.g., Cu, Cd, Hg, Pb, Pt, etc.), exerting cellular protection against their toxicity [89,90,91,92,93,94].

One of the main interesting functions of MTs lies in their ability to protect cells and tissues against oxidative stress from multiples sources. MTs act as a potent antioxidant scavenging of free radicals, protecting the cells against the toxic effects from ROS and reactive nitrogen species (RNS) or electrophiles [95,96]. Under oxidative stress conditions, zinc is released from MTs and their free sulfhydryl groups may act as effective scavengers of free radicals. The antioxidant role of MTs has received broad experimental support both through in vitro and in vivo studies, using wild type and genetically modified animal models [97,98]. Furthermore, MTs can protect the cell by sequestering copper or iron, preventing their participation in redox reactions, as the Fenton’s one, and consequently avoiding the release of free radicals [99,100]. Alternatively, MTs may function as an indirect antioxidant by providing metal cofactors for antioxidant enzymes such as SOD, among others [101].

Concerning their neuroprotective role, the importance of MTs in the central nervous system (CNS) underlies in the brain high oxygen consumption rates and its elevated zinc levels, which makes it susceptible to oxidative stress and must tightly regulate the zinc homeostasis, respectively. Within the CNS, MT1/2 isoforms are primarily expressed in astrocytes and to a much lower extent in neurons. Moreover, the cells of microglia and oligodendrocytes, lacking expression of MTs under normal conditions, show up-regulation of these proteins in response to brain injury [102,103,104,105]. In relation to the MT3, while there are contradictory reports about its expression, it is generally accepted that is mainly localized in neural cells, especially those with high concentrations of zinc. Intriguingly, this MT3 protein was identified for the first time in patients with Alzheimer’s disease as a neuronal growth-inhibitory factor [82]. Its growth-inhibitory activity seems to be a specific functional characteristic of this MT3 isoform [104,106,107,108,109]. In contrast to MT1/2, which are up-regulated even against small damages, the response of MT3 to damage does not provide the same pattern in response to brain injury according to in vivo studies [110]. Since the neural retina is considered part of the CNS, the MTs perform within the eye complementary functions to those previously described. Neuroprotection mediated by MTs can be partially attributed to intracellular free radical scavenging and to zinc and copper regulation in injured cells [111,112]. Furthermore, this redox-induced zinc ions release activates multiple zinc-dependent transcription factors and proteins involved in MT-triggered neuroprotective processes [98,112,113,114]. There are also evidences of the extracellular role of MTs, released from astrocytes, in neuronal survival as well as enhancing regenerative growth in the injured brain [110,115,116]. The damage response mediated by extracellular MTs involves the membrane receptor protein megalin (LRP2), which belongs to the family of low-density lipoprotein receptor-related proteins. LRP2 binds and internalizes MTs in the neuronal cytoplasm, which activates the signal transduction pathways that support neurite outgrowth and survival [116,117,118,119,120].

Finally, the MT isoforms play an anti-apoptotic protective role [121,122]. Given that the regulation of the intracellular zinc concentration is essential for cell survival, a decrease of intracellular zinc levels or an increase in cytosolic free zinc can lead to apoptosis [123,124,125]. MTs supply zinc or other metals to target molecules, including enzymes, transcription factor or tumor suppressor gene products [126,127,128,129,130]. Furthermore, MTs interact with some proteins involved in apoptosis, and the specific isoforms MT1 and MT2 regulate the level, activity and cellular location of NF-κB, a transcription factor that is involved in the regulation of cell death [131,132,133]. This makes MTs to play an important role in the process of cancer initiation and progression as well as in the response of patients to specific treatment [134,135].

#### 3.1.3. The Zn-MT Redox Cycle as an Antioxidant Defense Mechanism

Under oxidative stress conditions, MTs act as effective scavengers of free radicals through the Zn-MT redox system. This redox cycle, simplified in Figure 3 (panel b), has been previously described in numerous publications to date [71,87,98,136,137,138,139]. In brief, when the cellular environment becomes oxidized, zinc bound to MT (Zn-MT) is released through oxidation of the thiolate cluster with the consequent formation of MT-disulfide (thionin) [140]. This process is enhanced in the presence of free radicals such as nitric oxide, ROS and GSSG. The released zinc can be stored (zincosomes), transferred to other zinc-binding proteins or interact with transcription factors, including the metal regulatory transcription factor 1 (MTF-1), which induces MTs expression in the cellular nucleus [74]. MT-disulfide is unstable and may be degraded. However, when the environment becomes reduced due to, for example, an increase in the glutathione/glutathione disulfide ratio (GSH/GSSG), MT disulfide is reduced to MT-thiol (thionein), process that may be facilitated by a selenium-derived catalyst [141]. Later, in the presence of zinc, the Zn–MT system will be then quickly reconstituted to its reduced form [142].

#### 3.1.4. MTs Regulation

Despite the widely conserved sequence of MTs, the RNA expression level of each MT isoform is unique mainly due to differences in the number, location, and orientation of the regulatory sequences of their promoters [143]. Furthermore, human MT isoforms are regulated in a cell type–specific manner, which may allow each isoform to have a specific role [144,145]. The basal activity of human MTs is mainly regulated by several general transcription factors, including the transcription factor II D (TFIID) complex, which consists of the TATA-binding protein (TBP) and TBP-associated factors, and the transcription factor Sp1, which binds to multiple GC boxes [143,146,147]. In addition, while MT1 and MT2 expression can be induced by a wide variety of stimuli, including metal ions, inflammatory mediators (such as cytokines), growth factors, hormones, oxidative stress, irradiation, and other stressors, MT3 and MT4 are constitutive expressed tissue-specific isoforms that do not respond to metal induction [72,148,149]. Induced expression of the MT1 and MT2 genes occurs primarily at the level of transcription through regulatory sequences in the 5′-promoter region of MTs genes [110,150,151]. These cis-acting DNA elements that can bind diverse transcription factor include:

Article I. Metal response elements (MRE): The MTF-1 transcription factor is a central regulator of the metal inducible expression levels of MT1 and MT2. The binding of zinc to MTF-1 enables its union to MREs in the promoter region, which initiates gene transcription [146,147]. Heavy metal ions like Zn, Cu, Cd or Hg, as well as hypoxia, oxidative stress, glucocorticoids, nitric oxide and high temperature induce the transcriptional activity of MTF-1 [152,153,154,155].
Article II. Antioxidant (or electrophile) response element (ARE): ROS and oxidative stress increase MT1 and MT2 expression in a dose-dependent manner [156,157], which, in addition to an ARE in the promoter region, involves ARE-binding transcription factors, as well as MTF-1 [158,159].
Article III. Glucocorticoid response elements (GREs): Glucocorticoids like corticosterone and dexamethasone induce MT1 and MT2 expression through GREs [147,160,161]. In humans, the induction of MT2A by glucocorticoids is significantly higher than the MT1 isoforms [144].
Article IV. STAT-binding sites: Elements activated by signal transducers and activators of transcription (STAT) proteins mediate transcriptional responses to cytokines [162]. Pro-inflammatory cytokines secreted by the activated macrophages during acute inflammation induce the expression of MTs [163]. STAT proteins convert the cytokine signal into gene expression programs regulating the inflammatory response in different cell types [164,165] and, in a cooperative mechanism of negative feedback, MTs inhibit the release of pro-inflammatory cytokines [166,167].
Article V. MTs promoters include other binding sequences for transcription factors such as Sp1, the activator proteins 1 and 2 (AP-1 and AP-2), the upstream stimulatory factor 1 (USF1) and the nuclear factor 1 (NF-1), which are regulators of the MTs inducible expression [158,159,168,169].

On the other hand, some transcription factors and DNA binding proteins have been reported as down-regulators of MTs expression, including transcription factor PU.1 [170,171], the 120-kDa zinc finger protein (PZ120) [172], the CCAAT/enhancer-binding protein alpha (C/EBP α) [173], and the Ku protein [174], among others. Moreover, epigenetic modifications can also modulate MTs transcription, such as the methylated and un-methylated MT1 promoters that are differentially regulated by DNA methyltransferase and methyl-CpG binding proteins [175]. Also, acetylation of histone binding sites results in chromatin unfolding and transcription induction [176]. Finally, the levels of MTs are influenced by the intracellular protein degradation, in which MTs turnover depends on cellular conditions. Oxidized and/or metalated proteins are more resistant to degradation that the native ones, directly depending on the metal and occupied binding sites [177,178].

### 3.2. Oxidative Stress, Age-Related Diseases and MTs in the Human Eye

As previously mentioned, the excess of ROS and subsequent oxidative stress causes damage to DNA, lipids and proteins inhibiting their biological functions [179,180,181,182,183], which affects the eye natural barriers against oxidative damage resulting in the pathogenesis of numerous age-related ocular diseases including cataracts [184,185,186], photokeratoconjunctivitis and photokeratitis [187,188,189,190], dry-eye disorders [191,192,193,194,195,196,197,198], diabetic retinopathy [199,200,201,202,203,204,205,206,207,208,209], retinal vascular diseases [210,211,212], retinal dystrophies [213], glaucoma [214,215,216,217,218,219,220,221,222,223] and AMD [224,225,226,227,228,229,230,231,232]. Under normal physiological conditions, oxidative stress is reduced by the presence of antioxidants and cellular repair systems, being the ocular tissues enriched in MTs that undoubtedly contribute to mitigate the negative effects of oxidants by capturing and neutralizing free radicals through their cysteine sulfur ligands [79,138,233,234].

**Figure 4:**
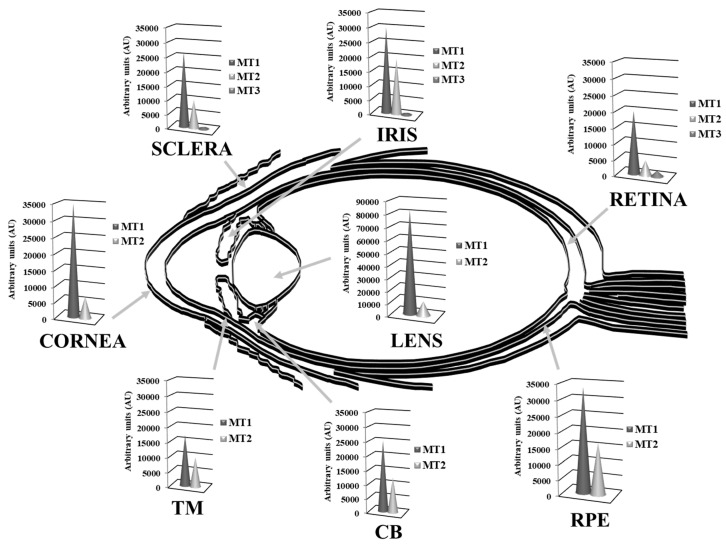
Gene expression profiling of MT1 (MT1A, MT1B, MT1E, MT1F, MT1G, MT1H, MT1M AND MT1X), MT2 (MT2A) and MT3 isoforms in human eye tissues, according to Alvarez et al., 2012 [15] and adapted, expressed in arbitrary units (AU) after normalization with internal controls. Scale bars of Y-axis range from 0 to 35,000 AU for each eye tissue, with the exception of lens, which Y-axis scale bar ranges from 0 to 90,000 AU.

Multiple MT isoforms are highly and diversely expressed in the human ocular tissues [15,138]. Alvarez et al., 2012 [15] analyzed the gene expression profile of MTs isoforms in normal human eye donors (cadavers) by microarray techniques and compared their abundance between tissues, showing in Figure 4 the MT isoforms expression levels within eye structures, clustered in MT1, MT2 and MT3 subgroups. While MT1 and MT2 groups are abundantly expressed, exceedingly in the cornea, lens and retina-RPE tissues, MT3 is barely distributed in the neurosensory retina. The MT1A, MT2A and MT1X sub-isoforms are highly present in all ocular tissues, while MT1E, MT1F, MT1M and MT1G are expressed in much lower levels, with the exception of MT1G that is abundantly distributed in lens followed by the RPE. Conversely, MT1H and MT3 isoforms are expressed in low levels, but restricted preferentially to the lens and the retina, respectively. Besides, according to this study, MT1 and MT2 protein isoforms are highly expressed in the epithelial and endothelial corneal cells and confined in the cytoplasm.

Using micro-dissected human lens epithelial cells and fibers, Oppermann et al. 2001 [235] analyzed the MT expression at mRNA level by reverse transcription polymerase chain reaction (RT-PCR) and at a protein level by immunoblotting, observing different spatial expression patterns. Specifically, MT1E, MT1F, MT1G, and MT1H isoforms were detected in both lens epithelium and fiber cells, while MT1E and MT1F isoforms were expressed at slightly higher levels in the epithelium than in the lens fibers. By contrast, MT2A was only detected in lens epithelium, although low amounts of MT2A were detected in fibers from individual lenses presenting very high amounts of RNA. Due to the low amplification specificity, spatial analysis was not performed on isoform MT1L. At protein level, a predominance of MT protein in the lens epithelium relative to the fibers was observed. These reported differences in the spatial expression patterns between MT1 and MT2 may indicate distinct roles for these isoforms in the human lens. According to Tate et al. 2002 [236], within the RPE and choroids, the MT isoforms were detected by in situ hybridization, and to a lesser extent, in the sclera. The RT-PCR analysis corroborated the in situ hybridization findings and demonstrated that MTs were mainly distributed in the RPE and the photoreceptor layer of the retina. Moreover, the high metabolic oxygen flux and high phagocytic activity of the RPE correlate with the high expression levels of MT isoforms and their protective function against apoptosis and oxidative stress [15,138].

Interestingly, MT3 isoform is preferentially distributed in the cytoplasm of the RGCs and the retinal nerve fiber layer (NFL), while is also localized in the stromal cells cytoplasm of the iris [138]. The cell-restricted and isoform-specific MT3 detection in RGCs may reflect biochemical and/or physiological differences with other MT isoforms. The neuronal RGCs are the first retinal cells to undergo cell death in age-related eye diseases such as glaucoma, and they are highly sensitive to damage during oxidative stress caused by intense visible and UV light exposure [217].

Therefore, the higher levels of expression and diversity of MT isoforms in human cornea, lens and retina-RPE suggest a MTs role in cellular defense against oxidative stress in these ocular tissues that represent natural barriers to external insults (i.e., UV light) with physiological roles against age-related eye diseases [237]. The high content of sulfhydryl groups allows MTs to scavenge superoxide anion and hydroxyl radical with an affinity more than 300-fold higher than that of reduced GSH [238]. The structure of MTs provides a chemical environment by which the cysteine sulfur ligands can be oxidized and reduced concomitantly with the release and binding of zinc (see Figure 3) [138,142], conferring the antioxidant activity on the Zn-MT complex [87,138]. The MT affinity for zinc ions varies depending on the number of elements bound per protein: the first four zinc ions bind tightly (log K = 11.8), the next two bind with intermediate strength (log K = 10), and the last one bind relatively weak (log K = 7.7) [71,77]. These different stability constants imply that zinc release is thermodynamically favorable in saturated MTs, which entails that Zn_7_-MT species present greater antioxidant capacity than Zn_x_-MT species (where x ranges from 0 to 6 zinc atoms), and so on [15,138]. Hence, the antioxidant activity and the capacity of MTs and the Zn-MT redox system of free radical scavenging might vary depending on the occupation rate of metals per protein molecule within each ocular protection barrier.

Therefore, the higher levels of expression and diversity of MT isoforms in human cornea, lens and retina-RPE suggest a MTs role in cellular defense against oxidative stress in these ocular tissues that represent natural barriers to external insults (i.e., UV light) with physiological roles against age-related eye diseases [237]. The high content of sulfhydryl groups allows MTs to scavenge superoxide anion and hydroxyl radical with an affinity more than 300-fold higher than that of reduced GSH [238]. The structure of MTs provides a chemical environment by which the cysteine sulfur ligands can be oxidized and reduced concomitantly with the release and binding of zinc (see Figure 3) [138,142], conferring the antioxidant activity on the Zn-MT complex [87,138]. The MT affinity for zinc ions varies depending on the number of elements bound per protein: the first four zinc ions bind tightly (log K = 11.8), the next two bind with intermediate strength (log K = 10), and the last one bind relatively weak (log K = 7.7) [71,77]. These different stability constants imply that zinc release is thermodynamically favorable in saturated MTs, which entails that Zn_7_-MT species present greater antioxidant capacity than Zn_x_-MT species (where x ranges from 0 to 6 zinc atoms), and so on [15,138]. Hence, the antioxidant activity and the capacity of MTs and the Zn-MT redox system of free radical scavenging might vary depending on the occupation rate of metals per protein molecule within each ocular protection barrier.

#### 3.2.1. Antioxidant Activity of MTs in the Ocular Surface

The production of oxidative stress might be induced on the ocular surface as a result of prolonged exposure to atmospheric oxygen and UV radiation, in addition to deficient support of antioxidants due to the instability in tear film, thereby, contributing to the onset of several eye diseases related with corneal defects. Tear fluid includes in its composition several antioxidants such as ascorbic acid, lactoferrin, uric acid, cysteine and MTs [239,240]. MTs are expressed in stem cells, located in the basal epithelium at the corneoscleral limbus [241,242], which are responsible of the maintenance of a healthy corneal epithelium. It has also been reported the MT expression in human corneas by laser capture microdissection [243] microarrays techniques [15] and RNA sequencing [244], where MT1A, MT2A and MT1X sub-isoforms were highly expressed [15]. In particular, immunohistochemistry analysis reflected that MT1/2 isoforms were confined in the cytoplasm of the corneal epithelium and endothelium [138]. Gottsch et al., 2003 [245] compared the gene expression profile in normal human corneal endothelium with corneal endothelium affected with Fuchs’ dystrophy using serial analysis of gene expression. These Fuchs’ endothelial cells exhibited a decrease in the abundance of transcripts related to antioxidants and proteins, conferring protection against toxic stress and an increase of MT2A mRNA, which may represent tissue injury repair in Fuchs’ dystrophy and/or tissue protection against oxidative stress. Conversely, De Roo et al., 2017 [246] also studied the transcriptome of corneal endothelial cells affected by Fuch’s dystrophy using microarrays and found that MT2A was down-regulated. These differences could be caused by different stages of the disease. They suggest that the oxidant-antioxidant imbalance in Fuchs’ endothelial cells could be a consequence of the down-regulation of antioxidants, including MTs, without compensatory up-regulation of catalase and glutathione-dependent antioxidants [247].

Recently, Kaluzhny et al., 2020 [248] evaluated the regulation of genes related to oxidative stress response in a reconstructed three-dimensional (3D) human corneal epithelial tissue, observing that only UVB led to significant up-regulation of MT3 while hydrogen peroxide, nitrogen-mustard and desiccating stress conditions have not exert any observable effect. Interestingly, mice injected with diquat capable to induce oxidative stress led to up-regulation of MT1 isoform [249], which may imply gene specific regulation mediated by oxidative stress. This cell- or tissue-specific antioxidant function of MTs was further discussed by a seminal work of Álvarez et al., 2012 [15]. Using an in vitro model of human corneal epithelial cells, the stoichiometry of the Zn-MT redox system was studied, showing that under steady-state conditions native MTs contained six zinc and one copper ions (Zn_6_Cu_1_-MT). Moreover, when zinc or pro-inflammatory cytokines were added separately, it favored the formation of Zn_7_-MT species, which may mean it interacts more effectively with ROS to decrease potential oxidative damage. Altogether, the number of zinc ions per MT molecule may predict the cell or tissue ability to overcome oxidative stress insults in the ocular surface of the human eye, with potential implication in the onset or progression of age-related ocular dystrophies.

#### 3.2.2. Antioxidant Activity of MTs in the Lens

The major threat to lens transparency is oxidative damage, especially from photooxidation origin, which accumulates over the lifetime of an individual [250] and is involved in the pathogenesis of cataracts [184]. The lens responds to oxidative stress by increasing the production of glutathione, SOD and CAT, according to classical literature [251,252]. However, during ageing the efficacy of the lens antioxidant defenses declines, with a rate of 12% per decade, contributing to the development of lens diseases [253]. Kantorow et al. 1998 [254] analyzed and compared, by differential display RT-PCR, the RNAs obtained from human lens epithelial cells of cataractous and control subjects, showing higher levels of MT2A associated with age related cataract. Studies by Whitson et al., 2017 [255] have shown that MT2A and MT1 are up-regulated in a GSH-depleted mouse model of cataracts, suggesting that they have a counteracting protective role. These findings support the hypothesis that the up-regulation of MT2A in the cataractous lenses represents a specific protective response of this tissue against oxidative or other stress events related with the onset of cataracts [254,256]. On the other hand, Hawse et al., 2003 [257] compared the global gene expression profiles between pooled age-matched human lens epithelium isolated from cataract and clear lenses (control subjects) by oligonucleotide microarray hybridization. The expression levels of a subset of genes altered were further evaluated by semi-quantitative RT-PCR, identifying significant decreases in genes associated with oxidative stress such as the MT1 isoforms in the cataractous human lens epithelium. All these findings suggested an age- and oxidative stress-related decrease in the antioxidant activity of MTs in the human lens.

In vitro studies reported that over-expression of MT2A in human lens epithelial cells results in protection against cadmium and tertiary butyl hydroperoxide-induced oxidative stress [258]. Later, using the lens epithelial cell line alphaTN4-1, the protective effects of MT1 and MT2 against UV-induced oxidative stress have been quantitatively determined [259]. Recent studies carried out by González-Iglesias et al., 2017 [260] reported that MT1 and MT2 isoforms are preferential located in the human lens epithelium by immunohistochemistry analysis. A human lens epithelial cell line was used as in vitro model to study the Zn-MT regulation and stoichiometry, under the native state and following zinc and pro-inflammatory interleukin-1α (IL1α) supplementation. Their findings suggested that, while Zn_3_Cu_0.2_MT species are present under control conditions, zinc or pro-inflammatory cytokines changed their elemental composition of MTs to Zn_7_Cu_0.1_MT or Zn_7_Cu_0.2_MT, indicating that the new MTs are saturated by zinc ions, rendering the cell more resistant to oxidative insults. It must be stressed that MT proteins in the lens are in different redox state, compared to the cornea, being the latter with the highest antioxidant power, which may be related to the roles of cornea and lens as the first, and second natural barriers combating free radicals, respectively [15,260].

#### 3.2.3. Antioxidant Activity of MTs in the Retina-RPE

The neurosensory retina and its RPE are particularly susceptible to oxidative stress because of their high rate of oxygen consumption, their exposure to visible and UV light and their high proportion of polyunsaturated fatty acids [261,262,263,264]. ROS are involved in several retinal pathologies, and therefore conscious strategies to enhance the production of antioxidant enzymes, including MTs, have been addressed to reduce oxidative stress or to promote cytoprotective-signaling pathways [213]. Seminal studies demonstrated that MT expression decreases in the retina-RPE with aging and oxidative stress, which results in release of zinc within RPE, most dramatically in the macular region [265]. Various evidence implicates the imbalance of the MT antioxidant defense system with the development of AMD as a result of RPE degeneration and photoreceptor loss [266]. Tate et al., 1993 [265] reported that MTs decline as a function of age in the macular-RPE of patients with AMD, with proportionately greater loss in the macula than in the periphery. Moreover, while the antioxidant CAT and the metal zinc also decrease in aged human retinal pigment epithelial cells with signs of AMD, like MTs, SOD seems to increase their total levels [267,268,269]. Recently, Wi et al., 2020 [270] observed higher expression of *MT3, MT1G, MT2A* and *GPX3* in Müller cells of the periphery of aged retinas compared with those cells of the foveal region using single-cell RNA sequencing, stating that the MT expression pattern may underlie foveal to peripheral retinal ageing gradient [271]. These observations triggered plenty of research both in animal models and in vitro cell cultures. Studies carried out on retinal and RPE homogenates from an early onset macular degeneration monkey model, detected a decrease in the activities of CAT and GPX antioxidants, lowered zinc concentration and reduced MT gene expression in affected retinas compared with control individuals [272]. Later, Miceli et al., 1999 [273] found that a zinc deficiency diet reduced the concentration of MT in the retina and the RPE, and increased the levels of retina lipid peroxidation in rats, where a crosstalk between oxidative stress and MT expression exists since MT transcription is up-regulated by oxidative stress [158].

Light-induced damage has been widely used as a model system to study retinal degeneration [274], whereby prolonged and/or raised light exposure causes retinal oxidative stress [20,43,275,276,277,278,279]. In this sense, Chen et al., 2004 [280] analyzed the gene expression profile following light-induced retinal damage in a mouse model. The microarray analysis of RNA isolated from mice retinas subjected to acute photic injury identified 70 genes up-regulated (≥2-fold) when compared with control subjects, determining the overexpression of MT1 and MT2 isoforms subsequently confirmed by qPCR. Similarly, the simultaneous analysis of MT at protein and RNA levels by immunoblot, immunohistochemistry and qPCR in light-induced oxidative damage mouse retinas confirmed the increased expression of the antioxidant MTs, and suggested that their up-regulation is an important acute retinal response to photo-oxidative stress [281]. The up-regulation of MT2A after light exposure was also found by Natoli et al., 2010 [282] in a rat model of retinal degeneration using microarrays. Later, Tsuruma et al., 2012 [283] evaluated the specific roles of MT1, MT2 and MT3 isoforms in light-induced retinal degeneration using MT1/2 deficient [284,285,286], MT3–deficient and wild-type (WT) mice. The expression levels of all MT isoforms were significantly up-regulated in the murine retinas after light exposure. The functional role of MTs in light-induced retinal dysfunction was investigated in the MT1/2- and MT3–deficient mice by electroretinogram recording, which a-waves and b-waves reflected the function of photoreceptors and Müller cells, respectively. Interestingly both reductions in the a- and b-wave amplitudes were significantly greater in the MT3–deficient mice than in the WT following light exposure. Moreover, the ONL was significantly thinner in the MT3-deficient mice compared to the light exposed WT, while no changes in the retinal function or ONL thickness were observed in the MT1/2–deficient mice. These observations were confirmed using MT3 knockdown by siRNA in a mouse transformed photoreceptor-cell line (661W) subjected to light-induced damage, observing amplified cell damage and increased production of ROS in response to light exposure [283]. Altogether, these findings suggested that MT3 might protect more effectively against light-induced retinal damage compared to MT1 and MT2, probably mediated by its antioxidant properties in addition to other major biological roles in cellular processes and diseases [287].

MT1/2-deficient mice were also used to study the functional roles of MTs in retinal damage induced by intravitreal injection of N-methyl-D-aspartate (NMDA), together with the evaluation of the protective role of zinc and vitamin D_3_ [288]. NMDA induces retinal cell damage by activation of NMDA receptor, which leads to intracellular Ca^2+^ influx, increases ROS that affect cell viability [289,290] and depletes the intracellular GSH that may decrease the intracellular capacity against ROS [291,292]. Whilst intravitreal NMDA increased the MT immunostaining in the inner retina of WT mice (NFL, INL and some of the RGCs), no MT labeling was detected in the retina of MT1/2-deficient mice. Conversely, at mRNA level, MT2 increased but MT1 and MT3 decreased following NMDA injection in the WT mice. The cell loss in RGC layer after NMDA injection was significantly higher in MT1/2-deficient mice compared to the WT, but no differences were observed in the inner plexiform layer thickness in both studied animal models. Moreover, intravitreous injection of zinc or vitamin D_3_ 24-h after NMDA injury increased inner retinal MT immunoreactivity, in line with previous reports [159,293], and significantly attenuated NMDA-induced RGC layer loss in WT mice, although zinc pretreatment did not protect against RGCs loss in MT1/2-deficient mice. These findings indicated that MT up-regulation, specifically the MT2 isoform, might play an important neuroprotective role against retinal oxidative damage.

The use of cell models for in vitro studies as simplified systems has also contributed to elucidate the molecular mechanism by which MTs protect against oxidative stress in the retina. Tate et al., 1999 [294] used an in vitro model of zinc deficiency through the primary culture of RPE cells in low zinc medium to identify a correlation between the concentration of MTs and the activity of antioxidant enzymes with the oxidative damage of reactive intermediates. As reported, while in low zinc cultured cells (0.55 µM zinc), MTs and CAT content decreased following oxidative stress, the cultured cells in adequate zinc levels (14 µM zinc) increased both MTs and CAT levels after oxidative stress damage. Thereby, zinc may stabilize both proteins by protecting their functional thiol groups from oxidative damage [295], along with the induction of MTs synthesis [296,297], which contain a high number of zinc-thiolate clusters able to scavenge free radicals [136]. In line with this, Tate et al., 2006 [298] tested the effect of different zinc physicochemical formulations (zinc acetate, zinc chloride, zinc cysteine and zinc sulfate) on the concentration of antioxidant proteins in a primary culture of human RPE cells. Increased CAT and GPX activities and glutathione and MT cellular content were observed after zinc supplementation with all tested formulations, highlighting greatest increase using zinc cysteine. In addition, the cytotoxic effects of H_2_O_2_ and t-butyl hydroperoxide were reduced after pretreatment with these zinc formulations, concluding the strong antioxidant protective capacity of MT proteins through the Zn-MT redox cycle. Even more, the MT antioxidant role was evidenced by the former studies of Lu et al., 2002 [243] showing that elevation of MT levels by transfection of RPE cells (D407 spontaneously immortalized cell line) protected against toxic levels of cadmium [299], heme- and iron-induced oxidation and UV light-induced apoptosis. In this regard, the combination of N-retinylidene-N-retinylethanolamine (A2E) and blue light permitted to study causative genes of retinitis pigmentosa using primary cultures of RPE cells under oxidative conditions, showing altered expression of aryl hydrocarbon receptor (AHR) and retinal outer segment membrane protein 1 (ROM1), but also other genes, including MTs [300]. Similarly, the effect of A2E-induced oxidative stress on human RPE cells determined relevant differences in gene expression and splicing events contributing to elucidate molecular mechanisms linking oxidative stress and retinal dystrophies [301].

Commercial ARPE-19 cells, a spontaneously immortalized cell line of human RPE have been widely used as in vitro model to study the cellular effects of oxidative stress. Several works have reported that ARPE-19 cells tend to be extremely resistant to oxidative stress [302,303,304,305]. Even though their capacity for H_2_O_2_ scavenging is not superior to other cell models, ARPE-19 cells are able to withstand a single initial dose (i.e., bolus addition) of 15 mM H_2_O_2_ before lysosomal rupture and subsequent induction of apoptosis occur [305]. Karlsson et al., 2013 [306] depicted that ARPE-19 cells contain very high basal levels of MTs, ferritin and heat shock protein 70 (HSP70), which can be easily up-regulated following specific stimulation with zinc, iron, or heat, respectively. These steady state high protein levels, specifically those of MTs, may be related with their high resistance to oxidative stress. Later studies of the same laboratory explored whether the up-regulation of the synthesis of MTs, ferritin and HSP70 could further increase the tolerance of ARPE-19 facing oxidative stress [307], while attenuation of MTs levels would affect cell susceptibility to free radicals. Therefore, decreasing MT levels using siRNA made cells more susceptible to oxidative stress, being statistically significant after 10 mM H_2_O_2_ following the conditions assayed. Although, treatments with 100 µM ZnSO_4_, 500 µM FeCl_3_ or 43 °C heat shock exposure increased the levels of MTs, ferritin or HSP70, respectively, this up-regulation did not improve the tolerance of ARPE-19 cells to H_2_O_2_. It must be stressed that these studies were carried out with 60 h-old cells at a considerably higher density, likely affecting their sensitivity along with an already reached maximum protection. Moreover, same treatments using different cell lines from non-ocular tissues with much lower basal levels of these proteins provided increased resistance to H_2_O_2_ stress and prevention of lysosomal membrane permeabilization [308,309,310,311,312]. In connection with the above, Choudhary et al., 2005 [313] reported that ARPE-19 treated with a thionein hexapeptide H-Lys-Cys-Thr-Cys-Cys-Ala-OH, which is a fragment of mouse liver MT1 [314,315] significantly protected against H_2_O_2_ and 4-hydroxynonenal (HNE)-induced mitochondrial depolarization and ameliorated H_2_O_2_ and HNE-induced apoptosis. Additional evidence about the antioxidant properties of MTs were collected in the recent work of Wang et al., 2020 [316] on ARPE-19 cells exposed to sodium iodate-induced oxidative damage. In this study, cells transfected with overexpression plasmids of MT2A and MT1G genes showed a higher cell survival rate than control cells after exposure to sodium iodate (10 µM for 18 h). Conversely, silencing of the same genes with siRNA was associated with an accelerated cell death after treatment. The same study also demonstrated that the small molecule D609 acts as an effective antioxidant in RPE cells partially due to the induction of MTs expression.

The in vitro production of free radicals using H_2_O_2_ provides high variability of induced oxidative stress due to the Fenton reaction, where iron catalyzes the conversion of H_2_O_2_ into hydroxyl radical intracellular turning out into a non-specific and unstable mechanism difficult to reproduce [317]. In this way, additional stressors have been widely used to elicit free radicals in vitro and test the antioxidant mechanism of MTs in the RPE. Recently, Ahmed et al., 2016 [318] studied the mitochondrial oxidative stress in ARPE-19 cells treated with paraquat and in the presence or absence of the neuroprotective xaliproden. Paraquat is an herbicide structurally similar to 1-methyl-4-phenylpyridinium ion (MPP^+^), which is converted within the cell into a free radical that interacts with molecular oxygen to form superoxide [319]. Using these reagents, the authors observed by qPCR an induction in the transcription of MT1 in the presence of paraquat (5.5-fold) or xaliproden (3.3-fold) and a synergistic effect when combined (10-fold increase). Albeit the induction of the antioxidant pathways was not sufficient to prevent the toxic oxidant effect on ARPE-19 cells, the increased stimulation of MT1 and other antioxidant genes by the treatment combined (paraquat and xaliproden) resulted in an increased survival of the cells.

Similarly, ARPE-19 cells treated with 8-hydroxy-2-(di-n-propylamino)-tetralin (8-OH-DPAT) in the presence of paraquat led to a significant increase of MT1 expression along with other antioxidant enzymes, including heme oxygenase 1 (HO1), NAD(P)H dehydrogenase quinone 1 (NqO1), CAT and SOD1 [320]. The 8-OH-DPAT, a 5-hydroxy-tryptophan 1a (5HT1a) receptor agonist with neuroprotective effects [321], increased the MT synthesis in a primary culture of astrocytes [322] suggesting that 8-OH-DPAT may protect ARPE-19 cells by the specific induction of MT1, HO1 and NqO1 [320]. Moreover, differentiated ARPE-19 cells treated with paraquat, 8-OH-DPAT or a combination of both (paraquat and 8-OH-DPAT) showed a potent induction of MT1 (27-fold), HO1 (2-fold) and NqO1 (45-fold) expression [320], but with the absence of SOD1 and SOD2 overexpression. In line with this, the authors carried out in vivo studies in the retinas of mice treated with 8-OH-DPAT, showing a significant increase in the expression of MT1 (2-fold), HO-1 (4-fold), NqO1 (1.7-fold) CAT (17-fold) and SOD1 (1.6-fold), while SOD2 remained constant, concluding that 8-OH-DPAT specifically induces antioxidants (e.g., MTs), protecting the RPE and the neural retina from mitochondrial oxidative stress.

The protective role of MTs against oxidative species, and their inherent mechanisms, were further explored by Rodríguez-Menéndez et al., 2018 [323,324] using an in vitro model of immortalized human RPE cells (HRPEsv). Oxidative stress was induced in HRPEsv cells using 2,2′-azobis(2-methylpropionamidine) dihydrochloride (AAPH) and quantified by fluorescent probes, the MTs were induced through zinc supplementation and the Zn-MT system was quantitatively determined by mass spectrometry using enriched isotopes and chemometrics-based calculations. Zinc at different concentrations (25, 50 or 100 μM ZnSO_4_) specifically induced Zn-MT synthesis (1.6-, 3.6- and 11.9-fold, respectively), while pre-treated cells with zinc followed by AAPH oxidative stressor significantly reduced the ROS levels when compared to AAPH-treated cells. In these studies, the authors simultaneously obtained the zinc to MT ratio to determine whether oxidative stress may affect the stoichiometry and therefore their antioxidant power. In these HRPEsv cultured cells, under steady state conditions a Zn_1.4_Cu_0.11_MT stoichiometry was obtained, while under 100 μM zinc pre-treatment and APPH treatment the MT binding sites were saturated by zinc, i.e., Zn_7.4_Cu_0.03_MT. Moreover, AAPH treatment decreased MT levels (0.4-fold) but increased the number of zinc atoms bound to MTs (Zn_2.07_Cu_0.17_MT), which may be related with the antioxidant capacity of the Zn-MT system. The authors hypothesized that saturated MTs (Zn_7_-MT) may have the greater antioxidant capacity in HRPEsv cells, since when an excess of zinc is added the MTs interact more effectively with ROS [234,260]. It was therefore demonstrated that the Zn-MT overexpression significantly attenuated the oxidative stress induced by free radicals, whence the stoichiometry of Zn-MT plays an important role in oxidative stress response regulating cellular metal homeostasis and transcription factors.

## 4. Conclusions

Oxidative stress affects all structures of the human eye, specifically the ocular surface, the lens and the retina, which are considered antioxidant natural barriers against external insults. The imbalance between ROS production and the neutralizing ability of the antioxidant defense results in an excessive oxidative milieu that contributes to the onset or progression of age-related eye diseases. Oxidative stress has been implicated in the pathogenesis of dry-eye, cataracts, diabetic retinopathy, retinal vascular occlusions, retinal dystrophies, glaucoma and AMD diseases, inter alia. The underlying ocular antioxidant defense is a complex system responsible of free radicals scavenging and is composed of a vast variety of compounds, including both, enzymatic and non-enzymatic molecules. The main enzymatic antioxidants constituent the natural barriers of the human eye, i.e., cornea, lens and retina, include SOD, GPX, CAT and PRDX as primary antioxidants and TKT, G6PD, GSS and selenoproteins as secondary antioxidants. These antioxidants are diversely expressed along eye tissues and contribute to endogenous and exogenous free radical scavenging.

Even if MTs are not broadly considered enzymatic antioxidants, their redox activity dependent on zinc release warrants reappraisal consideration. This low molecular mass family of proteins neutralizes free radicals through their cysteine rich sulfur ligands and maintains cellular zinc homeostasis. The biological and biochemical significance of the multiple MT isoforms within the eye is not totally understood. The lack of specific antibodies for MT1 isoforms makes it difficult to assess their potential cell-specific distribution throughout the eye. Nowadays, novel therapeutic approaches against oxidative stress involved in eye diseases explore the enhancement of antioxidant enzymes production, including MTs, to reduce free radicals or to promote cytoprotection. Furthermore, considering that MTs expression decreases with age and oxidative stress, boosting the MT system may contribute to slow down or stop eye tissues degeneration and disease progression.

In this sense, the stoichiometry of Zn-MTs is of fundamental importance to oxidative stress response and to transduce oxidative signals into zinc signals. The MTs stoichiometry has been studied in three in vitro models of eye cells, obtaining Zn_6_MT species in corneal epithelial cells, Zn_3_MT species in the lens epithelial cells, and Zn_1.5_MT in RPE cells under control conditions. The observed different Zn-MT redox state may be related to the roles of the cornea as the initial barrier protecting the inner ocular tissues against external insults including UV light, the lens as second barrier responsible of the refraction of light, and the RPE as third barrier reducing light scatter. Therefore, the MT antioxidant capacity seems different within each tissue, being probably higher in the cornea, compared to the lens and to the RPE, which is related to the requirement of a more effective antioxidant system in the outer layers exposed to a higher oxidative milieu. As a perspective, the Zn-MT redox system might be used as a therapeutic target to combat oxidative stress in the ocular tissues for the treatment of age- and oxidative-stress-related eye diseases, through its reactivation by MT synthesis induction, thionein system regeneration, or both. Moreover, its combination with the activation of additional enzymatic antioxidants within the eye may contribute to diminish free radicals and enhance population healthy ageing.

## Abbreviations

5HT1a	5-hydroxy-tryptophan 1a
8-OH-DPAT	8-hydroxy-2-(di-n-propylamino)-tetralin
A2E	N-retinylidene-N-retinylethanolamine
AAPH	2,2′-azobis(2-methylpropionamidine) dihydrochloride
AHR	Aryl hydrocarbon receptor
ALDH	Aldehyde dehydrogenase
AMD	Age-related macular degeneration
AP-1	Activator proteins 1
AP-2	Activator proteins 2
ARE	Antioxidant response element
AU	Arbitrary units
C/EBPα	CCAAT/enhancer-binding protein alpha
CAT	Catalase
CB	Ciliary body
CNS	Central nervous system
G6PD	Glucose-6-phosphate dehydrogenase
GADPH	Glyceraldehyde-3-phosphate dehydrogenase
GPX	Glutathione peroxidase
GREs	Glucocorticoid response elements
GSH	Glutathione
GSR	Glutathione reductase
GSS	Glutathione synthetase
GSSG	Glutathione disulfide
HNE	4-hydroxynonenal
HO1	Heme oxygenase 1
HRPEsv	Human RPE cells
HSP70	Heat shock protein 70
IL1α	Interleukin-1α
INL	Inner nuclear layer
LRP-2	Membrane receptor protein megalin
MPP+	1-methyl-4-phenylpyridinium ion
MRE	Metal response elements
MTF-1	Metal regulatory transcription factor 1
MTs	Metallothioneins
NF-1	Nuclear factor 1
NFL	Nerve fiber layer
NMDA	N-methyl-D-aspartate
NqO1	NAD(P)H dehydrogenase quinone 1
O2·	Superoxide anion
ONL	Outer nuclear layer
PPP	Pentose phosphate pathway
PRDXs	Peroxiredoxins
PZ120	120-kilodalton zinc finger protein
RGC	Retinal ganglion cell
RNS	Reactive nitrogen species
ROM1	Retinal outer segment membrane protein 1
ROS	Reactive oxygen species
RPE	Retinal pigment epithelium
RT-PCR	Reverse transcription polymerase chain reaction
SEL	Selenoproteins
SOD	Superoxide dismutase
SRXN	Sulfiredoxin
STAT	Signal transducers and activators of transcription
TBP	TATA-binding protein
TFIID	Transcription factor II D
TKT	Transketolase
TM	Trabecular meshwork
TXN	Thioredoxin
UV	Ultraviolet
USF1	Upstream stimulatory factor 1
WT	Wild-type
